# TasselNetV2+: A Fast Implementation for High-Throughput Plant Counting From High-Resolution RGB Imagery

**DOI:** 10.3389/fpls.2020.541960

**Published:** 2020-12-07

**Authors:** Hao Lu, Zhiguo Cao

**Affiliations:** Key Laboratory of Image Processing and Intelligent Control, School of Artificial Intelligence and Automation, Huazhong University of Science and Technology, Wuhan, China

**Keywords:** plant counting, real-time processing, wheat ears, maize tassels, sorghum heads, pytorch implementation

## Abstract

Plant counting runs through almost every stage of agricultural production from seed breeding, germination, cultivation, fertilization, pollination to yield estimation, and harvesting. With the prevalence of digital cameras, graphics processing units and deep learning-based computer vision technology, plant counting has gradually shifted from traditional manual observation to vision-based automated solutions. One of popular solutions is a state-of-the-art object detection technique called Faster R-CNN where plant counts can be estimated from the number of bounding boxes detected. It has become a standard configuration for many plant counting systems in plant phenotyping. Faster R-CNN, however, is expensive in computation, particularly when dealing with high-resolution images. Unfortunately high-resolution imagery is frequently used in modern plant phenotyping platforms such as unmanned aerial vehicles, engendering inefficient image analysis. Such inefficiency largely limits the throughput of a phenotyping system. The goal of this work hence is to provide an effective and efficient tool for high-throughput plant counting from high-resolution RGB imagery. In contrast to conventional object detection, we encourage another promising paradigm termed object counting where plant counts are directly regressed from images, without detecting bounding boxes. In this work, by profiling the computational bottleneck, we implement a fast version of a state-of-the-art plant counting model TasselNetV2 with several minor yet effective modifications. We also provide insights why these modifications make sense. This fast version, TasselNetV2+, runs an order of magnitude faster than TasselNetV2, achieving around 30 fps on image resolution of 1980 × 1080, while it still retains the same level of counting accuracy. We validate its effectiveness on three plant counting tasks, including wheat ears counting, maize tassels counting, and sorghum heads counting. To encourage the use of this tool, our implementation has been made available online at https://tinyurl.com/TasselNetV2plus.

## 1. Introduction

Plant counting runs through almost every critical stage in agricultural production spreading from seed breeding (Wiles and Schweizer, 1999; Mussadiq et al., 2015; Guo et al., 2018), germination (Baofeng et al., 2016; Primicerio et al., 2017), cultivation (Yu et al., 2013; Liu et al., 2018), fertilization (Vos and Frinking, 1997; Boissard et al., 2008), pollination (Guo et al., 2015; Lu et al., 2017a; Sadeghi-Tehran et al., 2017), to yield estimation (Nuske et al., 2014; Ghosal et al., 2019; Zabawa et al., 2019), and harvesting (Häni et al., 2019; Jin et al., 2019). It also plays an important role in phenotyping functional traits of plants because many traits of interest are quantity-related, such as density (Madec et al., 2019) and the number of leaves (Giuffrida et al., 2015). This task is typically addressed with manual efforts in traditional agriculture. Manual counting, however, is subjective, tedious, error-prone, labor-intensive and inefficient due to fatigue of humans. Indeed agricultural practitioners have tried to automate this task over past decades (McDonald and Chen, 1990; Gomes and Leta, 2012; Kamilaris and Prenafeta-Boldú, 2018). Unfortunately this goal is not that easy to achieve due to versatile varieties of plants and intrinsic/extrinsic variations in reality. An automated plant counting system therefore is often limited to a controlled environment or a certain application scenario such that manual counting still takes place in most regions of the world.

With the prevalence of low-end digital cameras, high-performance graphics processing units (GPUs) and effective deep learning-based technology, computer vision has received much attention in plant counting due to increased reliability and decreased costs. Plant counting has thus gradually shifted from traditional manual counting to vision-based automated solutions. The most popular solution in plant counting comes from the success of a widely-used object detection framework called Faster Region-based Convolutional Neural Network (Faster R-CNN) (Ren et al., 2015). Faster R-CNN leverages a so-called region proposal network to identify potential object locations specified by bounding boxes, then passes these boxes into a classifier to assign object labels and confidence scores, and finally suppresses overlapped boxes per the confidence scores with a non-maximum suppression operator. The population of plants can be easily inferred from the number of bounding boxes detected. Faster R-CNN has been substantially applied to plant science and agriculture engineering communities to, for example, estimate ear density (Madec et al., 2019), detect maize tassels (Liu et al., 2020), localize sweet pepper (Halstead et al., 2018), identify crop seedlings (Quan et al., 2019), etc. However, it is expensive in computation due to the use of high-capacity ImageNet-pretrained models (Deng et al., 2009), such as VGG-16 (Simonyan and Zisserman, 2014) and ResNet (He et al., 2016), especially when dealing with high-resolution images. To acquire sufficient spatial resolution, high-resolution imagery, unfortunately, cannot be avoided in modern plant phenotyping platforms such as unmanned aerial vehicles. The problem is that it is intractable to directly train/test high-resolution images with Faster R-CNN due to GPU memory limitation. It has been reported in Madec et al. (2019) that the maximum image size acceptable for training Faster R-CNN is about 500 × 500 pixels. To address this, pre-splitting images becomes a common practice during both training and inference, rendering inefficient image analysis. For instance, according to Madec et al. (2019), the inference of around 100 high-resolution images can take more than 1 h. Such inefficiency largely limits the throughput of phenotyping. In modern high-throughput plant phenotyping systems, it is important that an image analysis tool can process high-resolution images within a short period of time.

In this paper, we advocate another promising plant counting paradigm—object counting. Instead of detecting object bounding boxes, object counting directly regresses object counts from an image. This is a much direct way when only the population of objects is concerned. Indeed the transductive principle suggests never to solve a harder problem than the target application necessities (Vapnik, 1998)—estimating object counts does not have to localize where objects are. Compared with object detection, object counting has many appealing advantages, for instance: (i) cheap manual annotations: learning object counting models only requires dotted annotations, rather than more expensive bounding boxes annotations used in object detection; (ii) simplified network architectures: object detection generally builds on multi-scale architectures such as feature pyramid networks (Lin et al., 2017; Tan et al., 2019) that have extensive decoding stages, while object counting, especially for local count regression models (Lu et al., 2017c; Xiong et al., 2019a), only needs an encoder; (iii) robust to partially overlapping instances: object detection tends to under-estimate object counts due to the existence of non-maximum suppression where partially overlapping instances are likely to be suppressed, while object counting naturally takes overlapping instances into account during ground-truth generation; and (iv) light-weight computational requirement: a light-weight object counting model trained from scratch can deliver sufficiently accurate counting accuracy, while object detection models generally require ImageNet-pretrained models, with also large GPU memory consumption.

In fact, object counting is a long-standing topic in computer vision. It can at least date back to early 2000s when counting is still a by-product of face/pedestrian detectors (Viola and Jones, 2001; Dalal and Triggs, 2005). Object counting then is gradually accepted as an independent research topic after the first counting-by-regression approach (Chan et al., 2008) appears where the global object count can be regressed from an image. The idea of counting by regression is further amplified by Lempitsky and Zisserman (2010) who introduce the concept of the density map. The density map is generated from dotted annotations with Gaussian smoothing such that each pixel is assigned with a value that corresponds to the object density, which transforms counting into a dense prediction problem (Lu et al., 2019, 2020). It has become the basic building block for many object counting models (Chen et al., 2013; Arteta et al., 2014) including recent deep counting networks (Zhang et al., 2015, 2016; Sindagi and Patel, 2017; Li et al., 2018; Liu et al., 2020; Ma et al., 2019; Xiong et al., 2019b). Most state-of-the-art counting networks, however, are also inefficient due to the use of pretrained VGG-16, which hinders their applicability in high-resolution imagery in plant counting. In plant science community, many attempts have also been made for direct counting by regression (Giuffrida et al., 2015, 2018; Rahnemoonfar and Sheppard, 2017; Wu et al., 2019). In particular, in our previous work we propose TasselNet (Lu et al., 2017c), a counting network based on the idea of local count regression, to count in-field maize tassels, demonstrating that even a low-capacity network can achieve reasonably good counting accuracy. We remark that, the idea of local count regression is particularly suitable for counting plants, because this paradigm is robust to size variations of plants. Such robustness is important because a plant per se is a self-changing system such that its physical size varies over time. Xiong et al. (2019a) further extends TasselNet to TasselNetV2 and applies this new version to wheat spikes counting. We observe that TasselNetV2 turns out to be a generic tool for plant counting and even achieves comparable accuracy in crowd counting against state-of-the-art deep counting networks in computer vision. Unfortunately both TasselNet and TasselNetV2 are only implemented in a research-orientated software, i.e., MATLAB, making them infeasible for practical deployment^1^.

**Figure 1:**
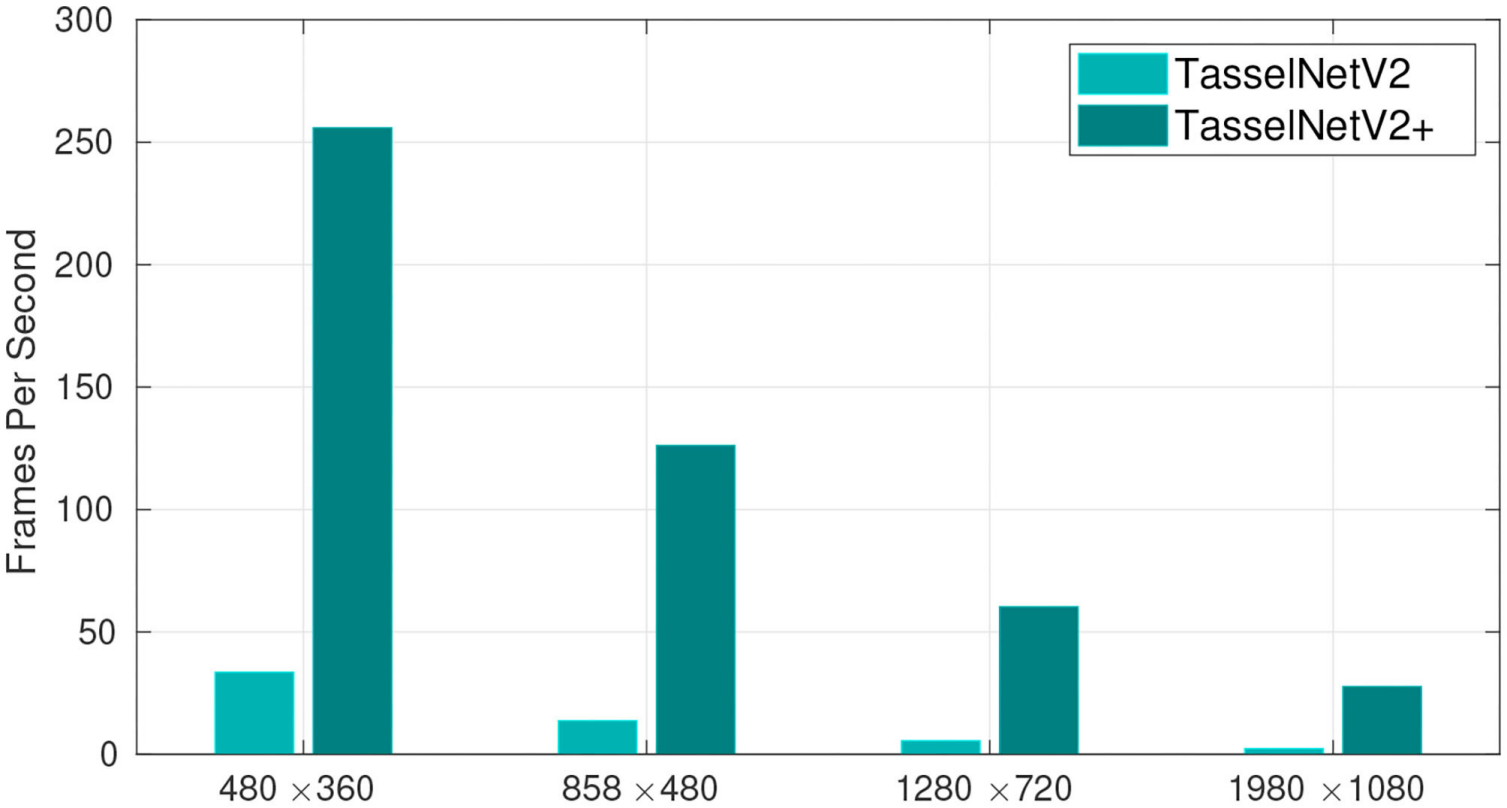
The number of processed frames per second with different image resolution. TasselNetV2+ is an order of magnitude faster than TasselNetV2. Frames per second are averaged over 100 independent trials on random input tested on GTX 1070 GPU, i7-8700 CPU, and 16 GB RAM.

In this work, we implement a fast version of TasselNetV2, TasselNetV2+, based on PyTorch (Paszke et al., 2019). By profiling the computational bottleneck, we make several minor yet effective modifications to TasselNetV2 to improve its efficiency. These modifications are based on a novel framework view of TasselNetV2, which decomposes TasselNetV2 into an encoder, a counter and a normalizer, allowing module-specific optimization and diagnosis. In particular, we find the main computational bottleneck of TasselNetV2 lies in the poor implementation of the normalizer. We address this issue with a novel mathematically-equivalent reformulation that enables an efficient GPU-based implementation. In addition, we notice a large portion of model parameters are included in the first convolutional layer of the counter, which also introduces many floating-point calculations. Inspired by a common practice in image classification (Lin et al., 2013; He et al., 2016), we make the same observation that the first convolutional layer of the counter can be safely replaced with global average pooling without performance loss. This simple modification significantly reduces model parameters, improves efficiency, and more importantly, enables flexible adaptation to different object sizes. Further, we also slightly improve the efficiency of the encoder by moving forward the last downsampling layer. Such a modification enlarges the receptive field (RF) by 17% so that extra context can be seen by the network. Altogether these modifications significantly improve the efficiency of TasselNetV2 by more than an order of magnitude, achieving around 30 fps on image resolution of 1980 × 1080 (tested on a low-end GTX1070 GPU), as shown in Figure 1. More importantly, these modifications have no negative effect on counting accuracy. To encourage the use of this tool, we has released our implementation online. We believe TasselNetV2+ will facilitate many counting-related tasks in plant phenotyping systems. In short, we make the following contributions:

- TasselNetV2+: a fast version of TasselNetV2 with significant optimization in efficiency;
- A framework view of TasselNetV2 as a concatenation of an encoder, a counter and a normalizer, which allows module-specific optimization and diagnosis;
- A novel reformulation of local-count normalization that enables an efficient GPU-based implementation.

## 2. Datasets and Methods

### 2.1. Plant Counting Datasets

Since the focus of this work is on the methodology part, we leverage three publicly available plant counting datasets in our evaluation.

The Wheat Ears Detection (WED) dataset was collected in France with a wheat field phenotyping platform using a Sony ILCE-6000 digital camera in 2017. Images were captured from a trial of 120 2 × 10 m microplots with 20 contrasting genotypes at 2.9 m distance to the ground. The image resolution was 6, 000 × 4, 000. The number of ears in each image varied from 80 to 170. The dataset included 236 images. 30, 729 wheat ears were identified and manually annotated with bounding boxes. More details about the dataset can be found in Madec et al. (2019).

The Maize Tassels Counting (MTC) dataset was collected from four experimental fields across China between 2010 and 2015 with 6 different maize cultivars. The images were captured from a 5-meter-height imaging device with a CCD digital camera (E450 Olympus). The image resolutions were 3648 × 2736, 4272 × 2848 and 3456 × 2304. The dataset had 361 images, with 186 training images and 175 testing images. The number of maize tassels varied from 0 to around 100. Each maize tassel was manually annotated with a single dot. More details can be found in Lu et al. (2017c).

The Sorghum Heads Counting (SHC) dataset was collected from a trail with 1440 plots in Australia during the 2015–2016 growing season. The images were captured using an unmanned aircraft vehicle at flight heights of 20 m and a flight speed of 3 m/s with a commercial RGB camera. The resolution of the camera was 5472 × 3648. In the released dataset, there were two subsets called “dataset1” and “dataset2” with 52 cropped images and 40 post-processed images, respectively. The cropped image resolution in dataset1 was 1154 × 1731. Forty processed images were of varied resolutions. These two subsets were chosen because only they were labeled with dotted annotations. More details can be found in Guo et al. (2018).

**Figure 2:**
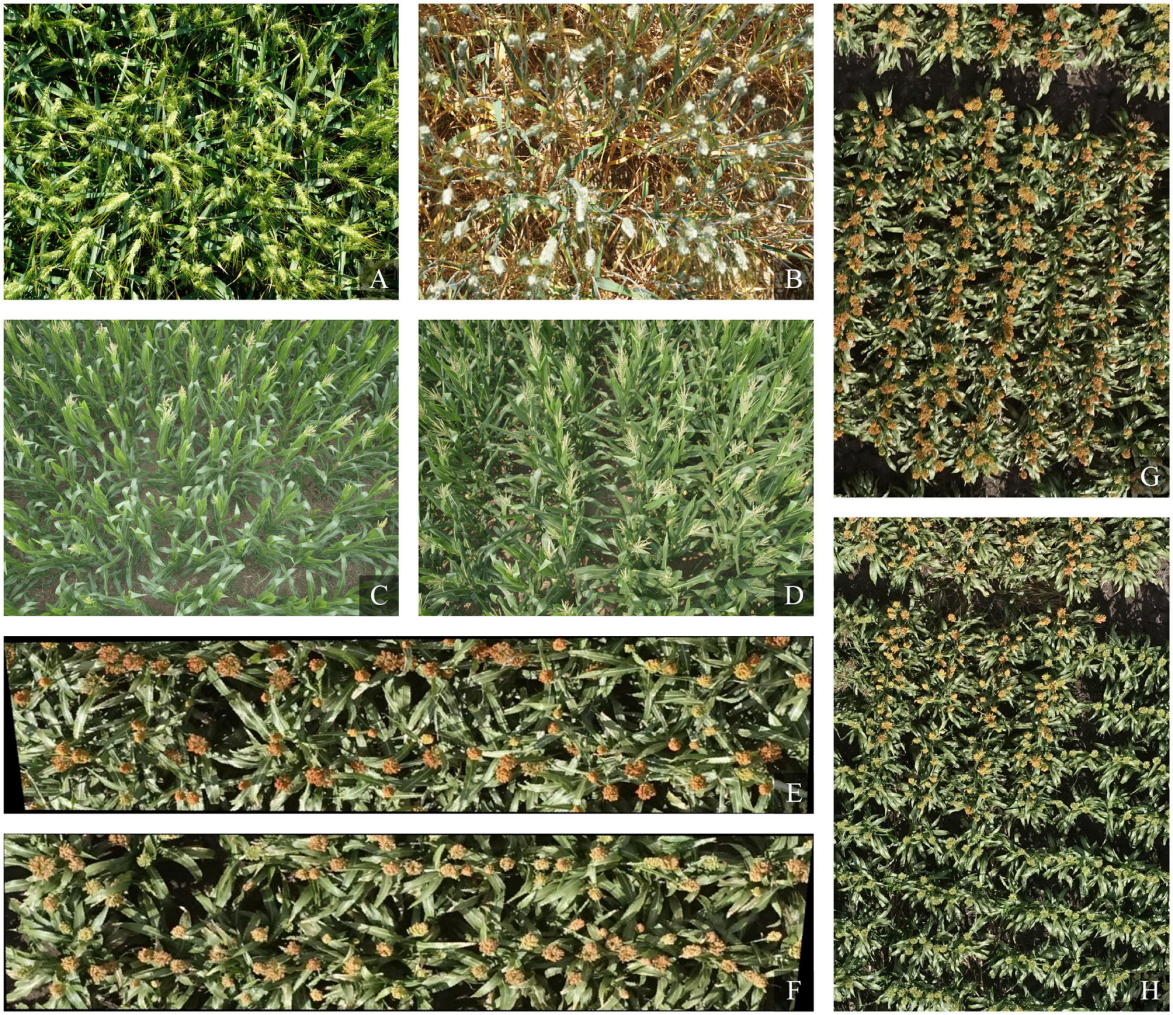
Example images on three plant counting datasets. Panels **(A,B)** are from the Wheat Ears Detection (WED) dataset, panels **(C,D)** are from the Maize Tassels Counting (MTC) dataset, and panels **(E–H)** are from the Sorghum Heads Counting (SHC) dataset.

Some example images of the three plant counting datasets are illustrated in Figure 2.

### 2.2. Recapping TasselNetV2

As the baseline of this work, here we first recap TasselNetV2 (Xiong et al., 2019a). TasselNetV2 extends TasselNet (Lu et al., 2017c)—the simplest implementation of local count regression, i.e., learning a mapping from local image features to local region counts. TasselNetV2 is inspired by an observation that the theoretical RF is wasted in TasselNet such that TasselNet is weak in modeling context. It addresses this issue by changing all fully-connected layers into convolutional ones to allow arbitrary sizes of input. Instead of sampling and operating on small image patches, TasselNetV2 processes full images. In this way, hidden RF can be freed to benefit some plant counting tasks where context is an important cue, such as wheat spikes counting (Xiong et al., 2019a).

**Figure 3:**
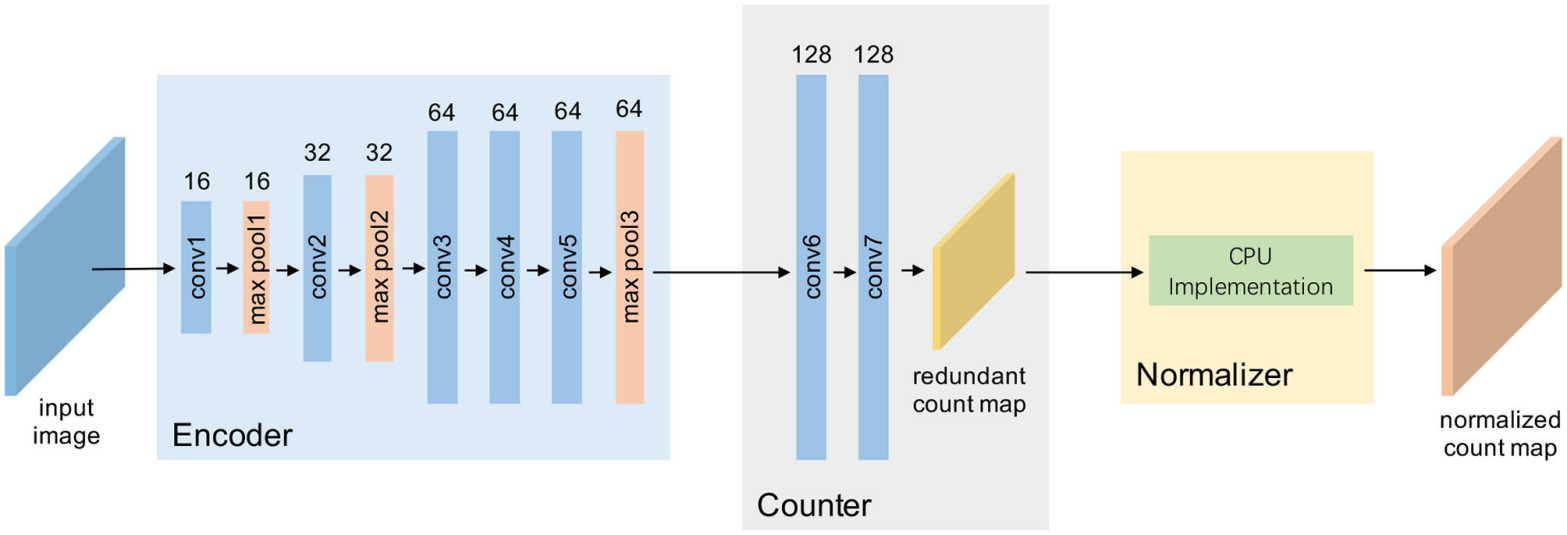
A framework view of TasselNetV2. Given an input image, TasselNetV2 processes it through an encoder with a few convolutional and downsampling layers, passes it into a counter to regress local counts, and finally, normalizes the local counts with a normalizer to generate the final output.

The network architecture of TasselNetV2 is shown in Figure 3. It includes 7 convolutional layers and 3 max pooling layers. Concretely, it is defined by **C**_3_(16)-**M**-**C**_3_(32)-**M**-**C**_3_(64)-**C**_3_(64)-**C**_3_(64)-**M**-**C**_8_(128)-**C**_1_(128)-**C**_1_(1), where **C**_*k*_(*m*) denotes a 2D convolutional layer with *m*-channel *k* × *k* filters, followed by batch normalization (BN) (Ioffe and Szegedy, 2015) and ReLU (Nair and Hinton, 2010), and **M** is a 2-stride max pooling operator with 2 × 2 kernel size. The last **C**(1) is the prediction layer where BN and ReLU are not included.

In local count regression, an image is mapped to a (redundant) count map where each local count in the count map corresponds to a *r* × *r* local region. The relative order between *r* and the output stride *s* determines whether the count map is redundant. Note that *r* ≥ *s*. The count map is redundant when *r* > *s*, because in this case every two adjacent local regions have a $\frac{r-s}{r}$ overlap. Only when *r* = *s* that the overlap disappears. According to the network definition above, *r* = 64 and *s* = 8 in TasselNetV2, so the resulting count map is redundant. A normalizer must follow for de-redundancy such that the sum of the final normalized count map can reflect the image count exactly. We call *r* × *r* the *base input size* of the network. The base input size is only related to the network architecture. This is a different concept from the input image size that can be arbitrarily large in theory. For example, given an input ***I*** ∈ ℝ^*r*×*r*×3^, TasselNetV2 defines a transformation *f* such that *f*(***I***):ℝ^*r*×*r*×3^ → ℝ; if ***I***′ ∈ ℝ^*H*×*W*×3^ where *H, W* ≫ *r* and are assumed to be divisible by *s*, then $f\left(I^{\prime }\right):{\mathbb{R}}^{H\times W\times 3}\to {\mathbb{R}}^{\frac{H}{s}\times \frac{W}{s}}$. This suggests the output size of the count map is irrelevant to the base input size when the input image size is larger than the base input size. We will use this concept extensively throughout this paper.

### 2.3. Profiling Computational Bottlenecks

Despite TasselNetV2 exhibits remarkable counting performance on counting maize tassels and wheat spikes (Xiong et al., 2019a), its efficiency does not meet the requirement of high-throughput high-resolution image analysis (Figure 1). It is thus natural to consider whether there is room for efficiency improvement. Before optimization, a prerequisite is to figure out where the computational bottleneck is.

From Figure 3, an important insight of this work is that, by decomposing the architecture, TasselNetV2 can be viewed as a concatenation of an encoder, a counter and a normalizer: the encoder specializes in encoding the image representation; the counter maps the image representation to the local count; and the normalizer normalizes redundant local counts and outputs the final image-level count. Such decomposition is essential to allow module-specific diagnosis and profiling.

**Figure 4:**
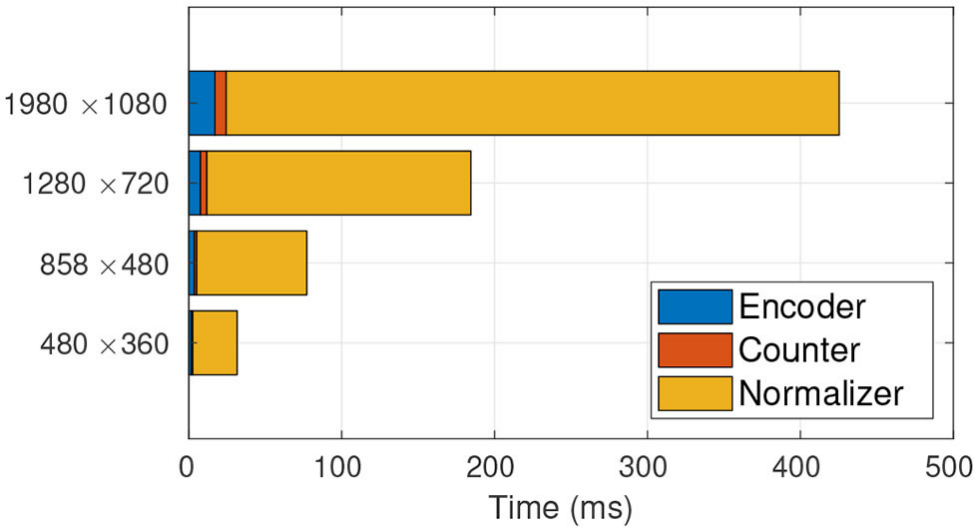
Time profiling of TasselNetV2. It is clear that the main computational bottleneck lies in the normalizer. From low resolution to high resolution, the normalizer takes up 91.72, 93.31, 93.62, 94.25% of the total processing time, respectively.

Given the framework view of TasselNetV2, we profile the time usage of each module in detail. The profiling results are shown in Figure 4. We surprisingly find that most of time consumption comes from the normalizer, and its occupancy even increases with increased image resolution. Since the bottleneck is found, the next step is to figure out why it wastes so much time. In what follows, we discuss this problem and our solution in detail.

### 2.4. Reformulating Local-Count Normalizer

**Figure 5:**
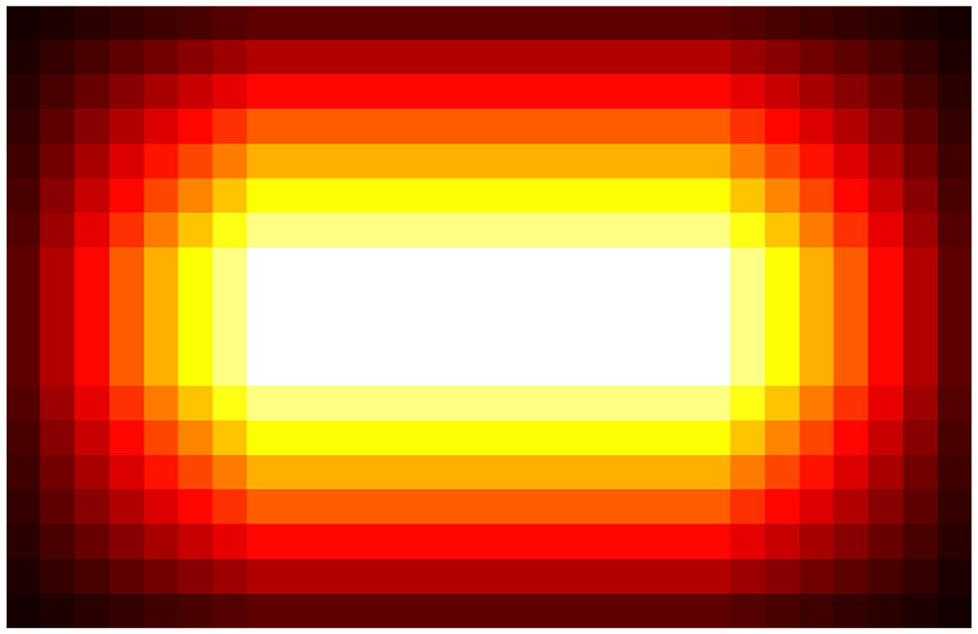
Visualization of redundancy. The lighter the color is, the more redundant the regions are. The redundancy gradually grows from border to center and then remains constant in central areas.

Let us first elaborate on how the normalizer works. As aforementioned, given an input image ***I*** ∈ ℝ^*H*×*W*×3^, TasselNetV2 produces a redundant count map $C_{r}\in {\mathbb{R}}^{\frac{H}{s}\times \frac{W}{s}}$. To remove redundancy, a normalizer is followed to generate a normalized count map $C_{n}\in {\mathbb{R}}^{H\times W}$. Notice that the spatial resolution is first reduced by *s* times and then recovered to the input resolution. TasselNetV2 achieves this by averaging each local count value *c* ∈ ***C***_*r*_ into to a *r* × *r* region, i.e., each element of the *r* × *r* region is assigned with an averaged count of $\frac{c}{r^{2}}$ (the sum of the local region still equals to *c*). By applying this rule to all local counts in ***C***_*r*_ and rearranging them following the same spatial order and the output stride, an upsampled count map $C_{u}\in {\mathbb{R}}^{H\times W}$ can be acquired. ***C***_*u*_ is still redundant. TasselNetV2 addresses this by constructing a reference map ***P*** ∈ ℝ^*H*×*W*^ that records how many times each location is counted. ***P*** can be an indicator of redundancy, as visualized in Figure 5. The final normalized count map $C_{n}\in {\mathbb{R}}^{H\times W}$ therefore can be computed by ***C***_*n*_ = ***C***_*u*_ ⊘ ***P***, where ⊘ denotes the element-wise division operator. Finally, the image-level count *c*_*I*_ can be computed by aggregating ***C***_*n*_, i.e.,

(1) $$\begin{array}{l} c_{I}=\overset{W}{\underset{x=1}{\sum }}\overset{H}{\underset{y=1}{\sum }}C_{n}\left(x,y\right), \end{array}$$

where ***C***_*n*_(*x, y*) is the value of ***C***_*n*_ indexed by *x* and *y*. The normalization process above can be implemented by Algorithm 1.

**Algorithm 1:**
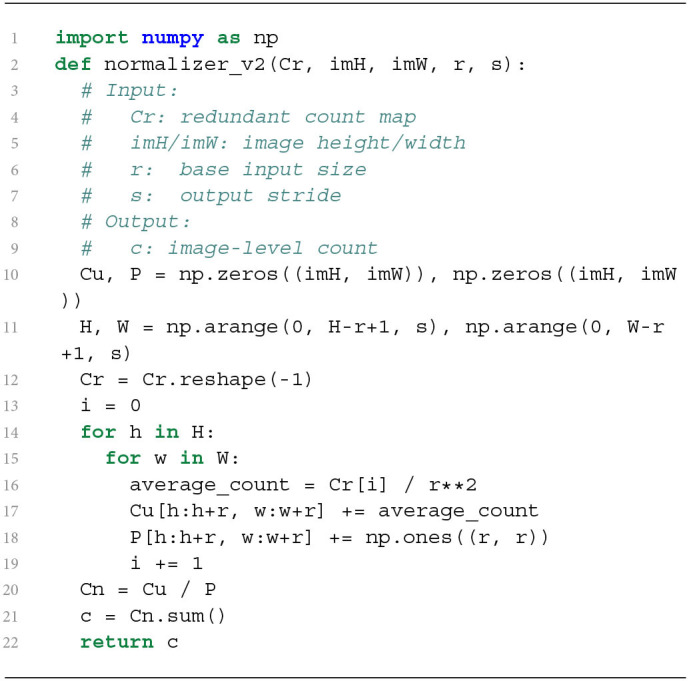
CPU implementation of redundant count normalization in TasselNetV2.

Algorithm 1 is a CPU-based sequential implementation. It is easy to verify that most time consumption takes place in the two nested for loops, leading to inefficient normalization. One possible solution may be to parallel this process with additional computational resources, while a more elegant way may be to pose the question: *Can we speed up the normalizer at the algorithmic level?* Our answer is *positive*. Our solution comes from a mathematically-equivalent reformulation of Equation (1), which takes the form

(2) $$\begin{array}{c} c_{I}=\overset{W}{\underset{x=1}{\sum }}\overset{H}{\underset{y=1}{\sum }}C_{n}\left(x,y\right)\\ \;=\overset{\frac{H}{s}\times \frac{W}{s}}{\underset{i=1}{\sum }}\overset{r}{\underset{x^{\prime }=1}{\sum }}\overset{r}{\underset{y^{\prime }=1}{\sum }}\frac{c_{r}\left(i\right)}{r^{2}\times P_{i}\left(x^{\prime },y^{\prime }\right)}\\ \;=\overset{\frac{H}{s}\times \frac{W}{s}}{\underset{i=1}{\sum }}c_{r}\left(i\right)\overset{r}{\underset{x^{\prime }=1}{\sum }}\overset{r}{\underset{y^{\prime }=1}{\sum }}\frac{1}{r^{2}\times P_{i}\left(x^{\prime },y^{\prime }\right)}, \end{array}$$

where $c_{r}\in {\mathbb{R}}^{\left(\frac{H}{s}\times \frac{W}{s}\right)\times 1}$ is the vectorized version of ***C***_*r*_, ***c***_*r*_(*i*) denotes the *i*-th local count of ***c***_*r*_, and ***P***_*i*_ indicates the *r* × *r* local region extracted from ***P*** that corresponds to ***c***_*r*_(*i*). The benefit of such a reformulation is that we can evade the explicit computation of ***C***_*u*_ and achieve per-region normalization simultaneously. In addition, ***P***_*i*_ can be efficiently constructed with modern image manipulation operators, such as im2col in MATLAB or fold in PyTorch. By defining another vector $q\in {\mathbb{R}}^{\left(\frac{H}{s}\times \frac{W}{s}\right)\times 1}$ where $q\left(i\right)=\overset{r}{\underset{x^{\prime }=1}{\sum }}\overset{r}{\underset{y^{\prime }=1}{\sum }}\frac{1}{r^{2}\times P_{i}\left(x^{\prime },y^{\prime }\right)}$, Equation (2) can be further simplified to

(3) $$\begin{array}{l} c_{I}=\overset{\frac{H}{s}\times \frac{W}{s}}{\underset{i=1}{\sum }}c_{r}\left(i\right)q\left(i\right)=c_{r}^{T}q. \end{array}$$

**Figure 6:**
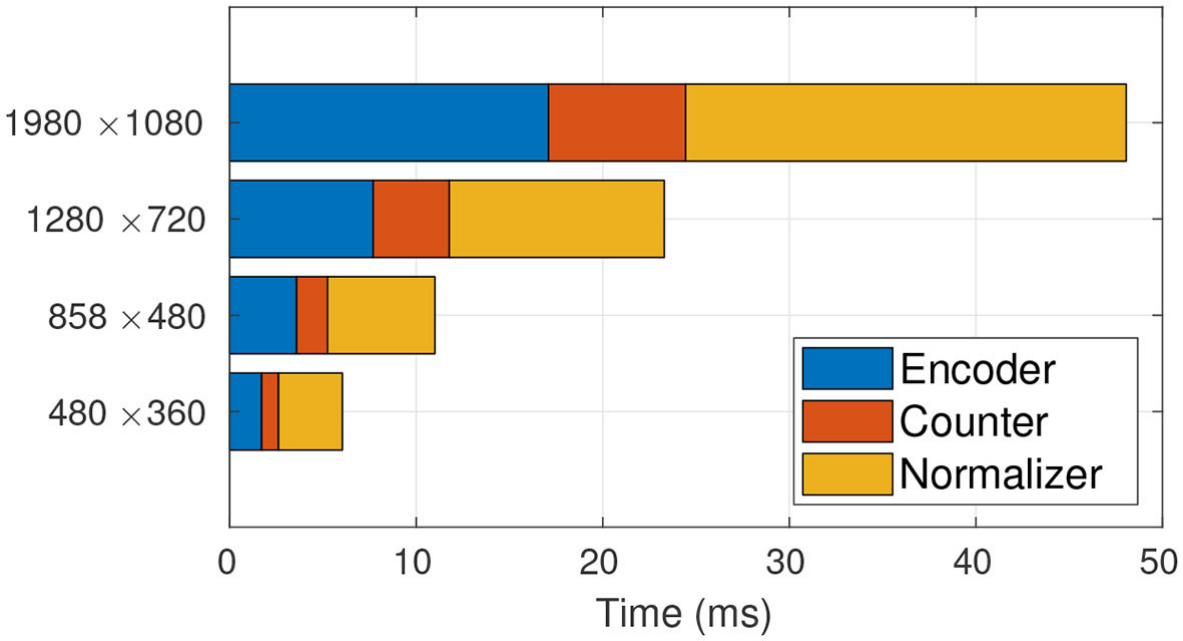
Time profiling of TasselNetV2 with the GPU-based normalizer. The GPU-based normalizer speeds up the inference significantly. From low resolution to high resolution, the normalizer only takes up 56.62, 52.36, 49.48, 49.15% of the total processing time, respectively.

This new formulation can be implemented by Algorithm 2. It is worth noting that Algorithm 2 is a full GPU-based implementation. We re-profile the time usage of TasselNetV2 with this new implementation. As shown in Figure 6, the time consumption of the normalizer reduces significantly.

**Algorithm 2:**
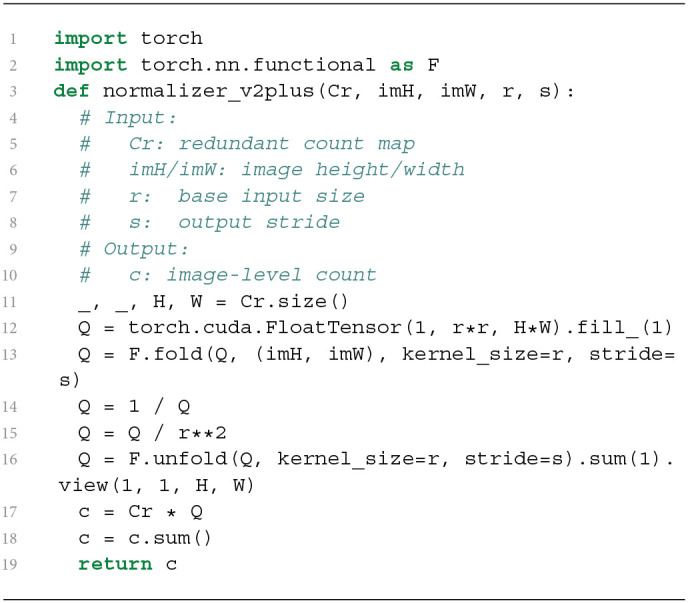
GPU implementation of redundant count normalization in TasselNetV2+.

### 2.5. Optimizing Encoder and Counter

After addressing the main computational bottleneck, we also take a closer look at the encoder and the counter to examine their possibility for further optimization. Indeed we find such possibility. For the counter, we notice that the number of parameter of the first convolutional layer is 8 × 8 × 64 × 128 = 524, 288, while the total number parameters of the model is 638, 993. That is to say, this single layer takes up 82.05% of model parameters. This fact motivates us to investigate the necessity of reserving such a parameter-extensive layer. Inspired by a common practice in image classification (Lin et al., 2013; He et al., 2016) where fully-connected layers are replaced with a global average pooling (GAP) layer, we apply this modification to TasselNetV2 and surprisingly find that almost no performance loss is observed (we will justify this point in section 3), which suggests the first convolutional layer in the counter can be safely replaced by GAP. Note that, the sense of “global” in GAP is relative to the base input size, rather than the input image size. It is still implemented by a standard average pooling layer, with the same kernel size compared to the size used in convolution, i.e., 8 × 8 for *r* = 64.

A very interesting property of introducing the GAP layer is that it allows flexible manipulation of the base input size *r* × *r* without changing the model complexity because GAP is a non-parametric layer. Allowing the change of *r* enables TasselNetV2+ to adapt to different object sizes in images. It is clear that, when resizing an image, object sizes change accordingly. *r* should also change to match the object size. For instance, if an image is upsampled by ×2, *r* also should be doubled. This is a hyper-parameter that needs to be tuned when choosing an appropriate image resolution in practice. Tuning *r* is easy in TasselNetV2+. Given the desired base input size *r* × *r* and the output stride *s*, one only needs to modify the kernel size of GAP to be $\frac{r}{s}$. Note that such a modification does not affect the model complexity. We will show later in section 3 how counting performance changes with changed base input sizes.

**Figure 7:**
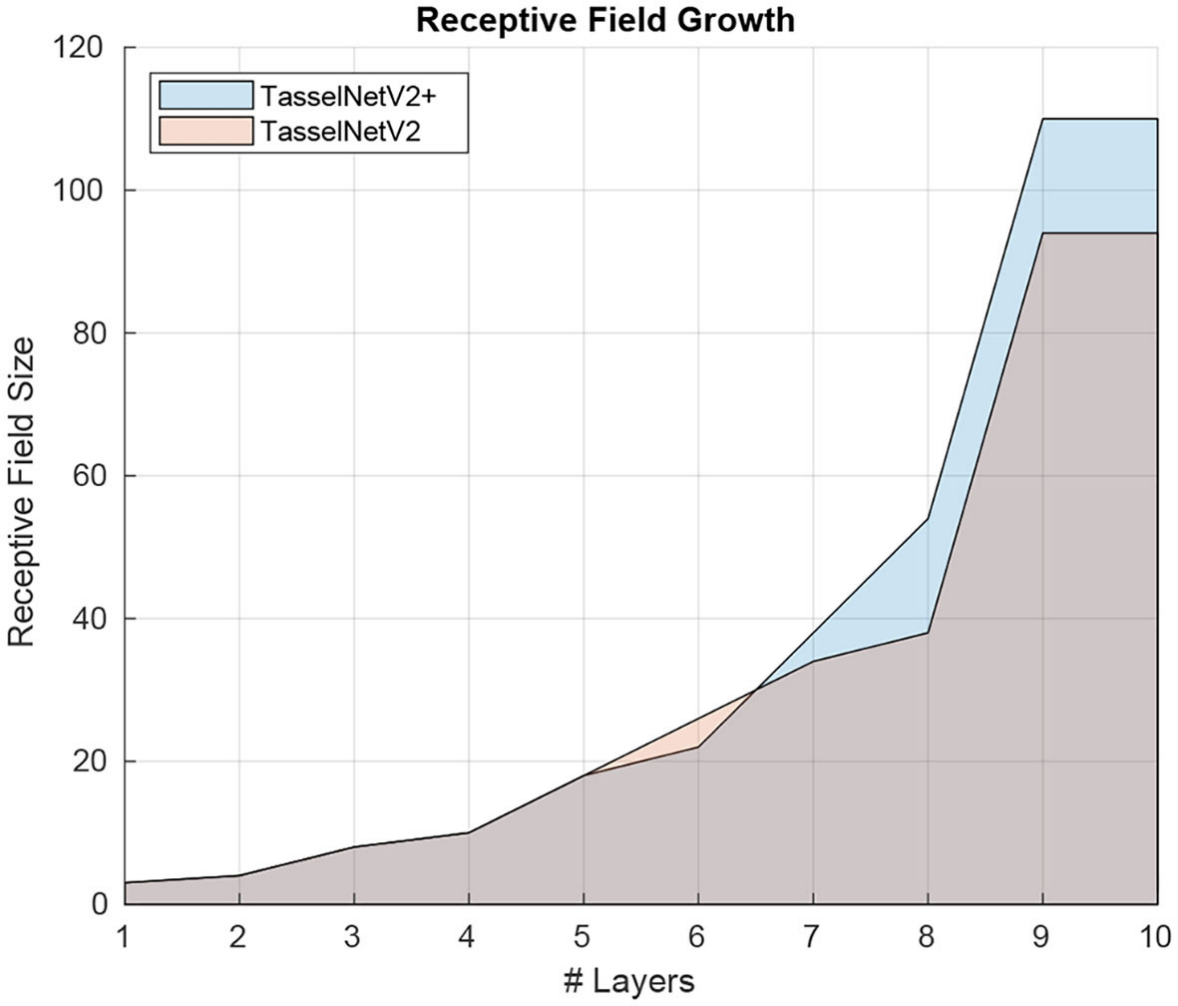
Receptive field comparison between TasselNetV2 and TasselNetV2+.

Regarding the encoder, it is not immediately clear on how to improve its efficiency because its design is sufficiently clean. Despite there exist efficient convolutional operators such as depthwise convolution, such efficiency still stays in theory, e.g., “depthwise convolution + pointwise convolution” used in MobileNet (Howard et al., 2017) is even less efficient than standard convolution in TasselNetV2 (23.76ms vs. 16.92ms for processing an 1920 × 1080 input with the encoder). Instead we find a simple trick that can improve the encoder efficiency. The trick is to move forward the last downsampling layer, right after the third convolutional layer. This simple modification leads to an efficiency improvement from 16.92 ms to 14.39 ms on an 1920 × 1080 input. The improvement can boil down to the early decrease of spatial resolution such that *conv4* and *conv5* are executed on low-resolution feature maps. The modification also increases the RF by 17%, from 94 to 110, as illustrated in Figure 7. The importance of RF for plant counting has been highlighted in Xiong et al. (2019a). Such increment of RF hence allows additional context modeling.

We remark that, since the improvements to the counter and the encoder are somewhat tricky and minor, we do not declare any novelty or contribution in this part.

### 2.6. Meeting TasselNetV2+

**Figure 8:**
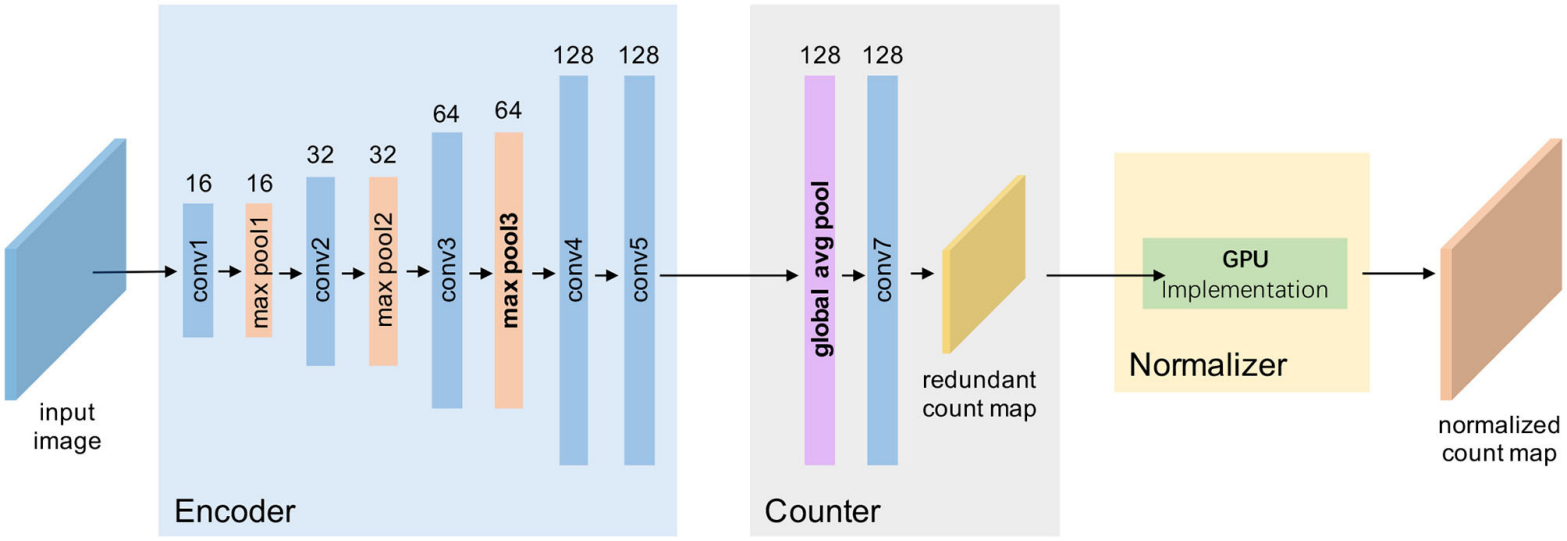
A framework view of TasselNetV2+. Our modifications are in boldface, including changing the downsampling behavior in the encoder, aggregating encoder features with global average pooling in the counter, and implementing a GPU-based normalizer.

Altogether the efficient normalizer, the trimmed counter, and the improved encoder construct a fast version of TasselNetV2 we call TasselNetV2+. Figure 8 highlights the improvements of TasselNetV2+ over TasselNetV2. Following the same notation in section 2.2, the architecture of TasselNetV2+ is formally defined by **C**_3_(16)-**M**-**C**_3_(32)-**M**-**C**_3_(64)-**M**-**C**_3_(128)-**C**_3_(128)-**A**_8_-**C**_1_(128)-**C**_1_(1), where **A**_8_ is the average pooling operator with 8 × 8 kernel size so that each inferred local count is still learned from a region of the base input size.

**Figure 9:**
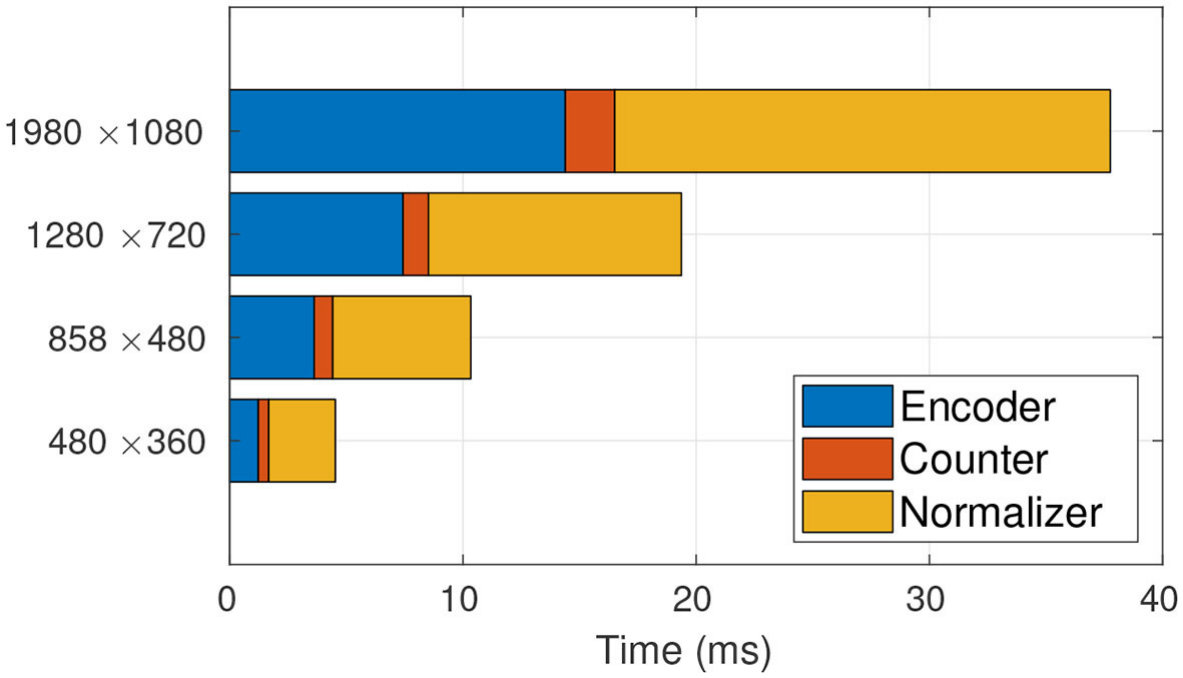
Time profiling of TasselNetV2+.

To showcase the overall effect in efficiency optimization, we again profile the time usage of TasselNetV2+ in Figure 9. It can be observed that, compared with Figure 6, the time consumption of the counter decreases significantly. Now TasselNetV2+ can process an 1920 × 1080 image in less than 40 ms. To give one a sense why TasselNetV2+ is significantly faster than TasselNetV2, we further summarize the number of parameters and GFLOPs (an indicator of the amount of floating-point operations) of two models. TasselNetV2 has 639K model parameters with the GFLOPs of 29.20, while TasselNetV2+ is with 262K and 12.42 GFLOPs (GFLOPs are based on an 1920 × 1080 input). Overall TasselNetV2+ is an order of magnitude faster than TasselNetV2 per Figure 1 with less parameters and GFLOPs. In section 3, we will show that the decrease of model parameters and GFLOPs does not imply the degradation of counting accuracy; instead TasselNetV2+ achieves almost the same counting accuracy compared to TasselNetV2.

## 3. Results and Discussions

The goal of this work is to provide an easy-to-use tool for plant counting and to improve the efficiency of TasselNetV2. Since the efficiency issue has already been justified in the previous sections, here we mainly address the concern on whether the increased efficiency comes at the cost of decreased accuracy. We evaluate TasselNetV2+ on three plant counting tasks, wheat ears counting (Madec et al., 2019), maize tassels counting (Lu et al., 2017c), and sorghum heads counting (Guo et al., 2018).

### 3.1. Wheat Ears Counting

Here we report results of TasselNetV2+, TasselNetV2 (Xiong et al., 2019a), TasselNet (Lu et al., 2017c), and Faster R-CNN (Ren et al., 2015) on the WED dataset (Madec et al., 2019). Since bounding boxes annotations are given, we only use the center points computed from bounding boxes to train TasselNetV2 and TasselNetV2+. We follow the same train/validation split used in Madec et al. (2019). We also follow (Madec et al., 2019) that designs a series of experiments with different downsampling rates of $\frac{1}{2}$, $\frac{1}{3}$, $\frac{1}{4}$, $\frac{1}{6}$, and $\frac{1}{8}$ and different cropped image sizes. This allows us to directly compare TasselNetV2+ with the results of Faster R-CNN reported in Madec et al. (2019). Note that, since in high resolution, the average size of wheat ears will be larger than the RF of TasselNetV2+, we also build several variants of TasselNetV2+ with changed base input sizes.

ℓ_1_ loss is used for training TasselNetV2 and TasselNetV2+. 256 × 256 or 512 × 512 image patches are randomly cropped from each image with random horizontal flipping for data augmentation (only one patch is sampled from each image in each epoch). The network is trained from scratch with a batch size of 8. Model parameters are initialized from the normal distribution with a standard deviation of 0.01. The stochastic gradient descent (SGD) optimizer is used for optimization. Parameters are updated for 500 epochs, with 10, 000 iterations. The learning rate is initially set to 0.01 and reduced by 10 × at the 200-th and 400-th epoch, respectively. The mean absolute error (MAE), root mean square error (RMSE), relative RMSE, and the coefficient of determination (*R*^2^) are reported.

Results are listed in Table 1. We can make the following observations:

- TasselNetV2+ achieves counting performance comparable to TasselNetV2 (5c vs. 5d);
- The best performance reported by TasselNetV2+ is slightly better than that reported by Faster R-CNN (4d vs. 1a), while TasselNetV2+ and Faster R-CNN achieve this at different resizing ratios ($\frac{1}{6}$ vs. $\frac{1}{2}$);
- Compared to Faster R-CNN (1a, 2a, 3b, and 4b), the performance of TasselNetV2+ is less sensitive to the change of image resolution (1b, 2b, 3d, and 4d). We believe the reason is that Faster R-CNN requires to encode sufficiently good appearance features to detect bounding boxes. In low image resolution, degraded appearance cues may lead to decreased performance of Faster R-CNN. By contrast, local count models like TasselNetV2 and TasselNetV2+ do not require detecting bounding boxes but work by counting repetitive visual patterns. Such repetitive patterns do not have to be the whole ear and instead can be any representative part of an ear. The patterns are not likely to change significantly with changed image resolution;
- Local regression models like TasselNetV2 and TasselNetV2+ generally work well when the ear size is small (4c, 4d, 5c, and 5d). This can be a valuable property in practice because these models make it possible for large-scale phenotyping from the sky, e.g., with unmanned aircraft vehicles, where the phenotyped plants often appear to be small in images;
- The counting performance of TasselNetV2+ improves when the base input size is larger than the average ear size (2b vs. 2c vs. 2d and 1b vs. 1c vs. 1d), which means the RF of the network should be large enough to cover the objects counted. In high resolution, the performance of TasselNetV2+ slightly decreases. We think the reason is that TasselNetV2+ is not sufficiently deep (with only 5 convolutional layers), the feature representation may not be encoded well at the high resolution (details of ears are rich in high resolution).
- Compared to Faster R-CNN, TasselNetV2+ is also efficient. It is reported in Madec et al. (2019) that the inference of Faster R-CNN on the $\frac{1}{2}$ resolution requires about 1 h to iterate over the validation set, while TasselNetV2+ only takes a few seconds.

**Table 1:** Performance on the Wheat Ears Detection dataset.

**Setting**	**Method**	**Resize**	**Base input size**	**Average ear size (pixels)**	**Cropped image size**	**MAE**	**RMSE**	**rRMSE**	***R*^2^**
1a	Faster R-CNN	1/2	–	110.8	500 × 500	**4.55**	5.94	5.30%	0.91
1b	TasselNetV2+	1/2	64	110.8	512 × 512	9.36	11.62	8.73%	0.67
1c	TasselNetV2+	1/2	128	110.8	512 × 512	7.45	9.52	7.47%	0.79
1d	TasselNetV2+	1/2	192	110.8	512 × 512	7.95	9.99	7.34%	0.75
2a	Faster R-CNN	1/3	–	73.9	500 × 500	–	–	5.40%	0.85
2b	TasselNetV2+	1/3	64	73.9	512 × 512	7.09	8.99	6.91%	0.81
2c	TasselNetV2+	1/3	96	73.9	512 × 512	6.13	7.58	5.88%	0.86
2d	TasselNetV2+	1/3	128	73.9	512 × 512	5.97	7.32	5.62%	0.86
3a	Faster R-CNN	1/4	–	55.4	250 × 250	–	–	11.20%	0.83
3b	Faster R-CNN	1/4	–	55.4	500 × 500	–	–	24.70%	0.87
3c	TasselNetV2+	1/4	64	55.4	256 × 256	6.03	7.53	5.71%	0.87
3d	TasselNetV2+	1/4	64	55.4	512 × 512	5.65	7.06	5.52%	0.88
3e	TasselNetV2+	1/4	96	55.4	512 × 512	5.29	6.71	5.26%	0.89
4a	Faster R-CNN	1/6	–	36.9	250 × 250	–	–	11.20%	0.75
4b	Faster R-CNN	1/6	–	36.9	500 × 500	–	–	38.50%	0.33
4c	TasselNetV2+	1/6	64	36.9	256 × 256	5.02	6.19	4.84%	0.91
4d	TasselNetV2+	1/6	64	36.9	512 × 512	4.59	**5.66**	**4.24**%	**0.92**
5a	Faster R-CNN	1/8	–	27.7	250 × 250	–	–	30.30%	0.62
5b	TasselNet	1/8	32	27.7	256 × 256	6.83	8.29	7.10%	0.79
5c	TasselNetV2	1/8	64	27.7	256 × 256	4.85	5.94	4.50%	0.91
5d	TasselNetV2+	1/8	64	27.7	256 × 256	4.93	6.08	4.63%	0.91

*The best performance is in boldface*.

### 3.2. Maize Tassels Counting

Here we evaluate TasselNetV2+ on the MTC dataset (Lu et al., 2017c). Following the same practices in Lu et al. (2017c) and Xiong et al. (2019a), we downsample images to its $\frac{1}{8}$ resolution for a fair comparison. We also report performance of TasselNetV2 and other state-of-the-art methods that have reported their counting performance on this dataset.

We follow the same training configuration used in the counting wheat ears except that, 256 × 256 image patches are randomly cropped, the batch size is set to 9 (with the same 10, 000 iterations). The MAE and RMSE are used as evaluation metrics. We also report *R*^2^ for TasselNetV2 and TasselNetV2+.

Results are shown in Table 2. It is clear that TasselNetV2+ performs no worse than TasselNetV2 and other state-of-the-art methods, with the best MAE of 5.1 and a comparable RMSE of 9.0. The slightly improved performance compared to Xiong et al. (2019a) may boil down to the improved training protocol (we observe that mini-batch training leads to more stable training behavior than single-image training used in Xiong et al., 2019a).

**Table 2:** Performance on the Maize Tassels Counting dataset.

**Method**	**MAE**	**RMSE**	***R*^2^**
Lu et al. (2016)	24.2	31.6	–
Lu et al. (2015)	19.6	26.1	–
Tota and Idrees (2015)	19.7	23.3	–
Lempitsky and Zisserman (2010)	11.9	14.8	–
Oñoro-Rubio and López-Sastre (2016)	21.0	25.5	–
Lu et al. (2017c)	6.6	9.6	–
Liu et al. (2020)	5.4	9.6	–
TasselNetV2 (Xiong et al., 2019a)	5.4	**8.8**	–
TasselNetV2 (Our Re-implementation)	**5.1**	9.3	0.8870
TasselNetV2+	**5.1**	9.0	**0.8880**

*The best performance is in boldface*.

### 3.3. Sorghum Heads Counting

Here we evaluate TasselNetV2+ on the SHC dataset. The SHC dataset is introduced by Guo et al. (2018) where two subsets with 52 and 40 images are labeled with dotted annotations, respectively. Since two datasets are generated in different ways, we evaluate TasselNetV2+ on them independently. For the dataset1 with 52 images, 26 images are randomly sampled for training, and the rest for testing. For the dataset2 with 40 images, 20 images are randomly sampled for training, and the rest for testing. We do not downsample the images in both training and testing.

We also follow the same training configuration used in counting wheat ears except that, 256 × 1024 image patches are randomly cropped, and the batch size is set to 5. We report MAE, RMSE and *R*^2^.

Results are shown in Table 3. Again TasselNetV2+ and TasselNetV2 achieve comparable counting performance. It is worth noting that, both models are trained with a limited number of training samples (no more than 30), which implies that TasselNetV2+ is applicable to small sample sizes. The *R*^2^ on the dataset2 is slightly poor, but we notice most inferred counts on this dataset are sufficiently accurate. Since the number of testing sample is limited, the computation of *R*^2^ may be biased by some outliers shown in Figure 11.

**Table 3:** Performance on the Sorghum Heads Counting dataset.

	**Dataset1**			**Dataset2**		
**Method**	**MAE**	**RMSE**	***R*^2^**	**MAE**	**RMSE**	***R*^2^**
TasselNetV2	17.96	21.33	0.9578	**3.54**	5.00	0.6115
TasselNetV2+	**17.53**	**20.60**	**0.9587**	3.58	**4.78**	**0.6767**

*The best performance is in boldface*.

### 3.4. Further Discussions

As a summary of experiments above, we compare merits and drawbacks of Faster R-CNN, TasselNet, TasselNetV2, and TasselNetV2+ in Table 4. Faster R-CNN is accurate and has good multi-scale adaptation, but it becomes slow when scaling to high-resolution images due to large model capacity and high GPU memory consumption. TasselNet is a prototype of the plant counting model with only dotted annotations required. It points out a promising plant counting paradigm under resource-constrained conditions, but also leaves many problems unsolved. TasselNetV2 improves the accuracy and efficiency of TasselNet with the same model capacity, but still cannot tackle high-resolution images well. TasselNetV2+ inherits all the advantages of TasselNet and TasselNetV2 and is also scalable to high resolution. Despite TasselNetV2+ may not generalize well to multiple scales, we consider it a good candidate for plant counting.

**Table 4:** Characteristics of different methods.

**Method**	**Accuracy**	**Speed**	**Resolution scalability**	**Scale adaptation**	**GPU memory consumption**	**Model capacity**
Faster R-CNN	Good	Slow	Poor	Good	High	Large
TasselNet	Fair	Relatively slow	Poor	Poor	Low	Small
TasselNetV2	Good	Relatively fast	Fair	Poor	Low	Small
TasselNetV2+	Good	Fast	Good	Poor	Low	Tiny

**Figure 10:**
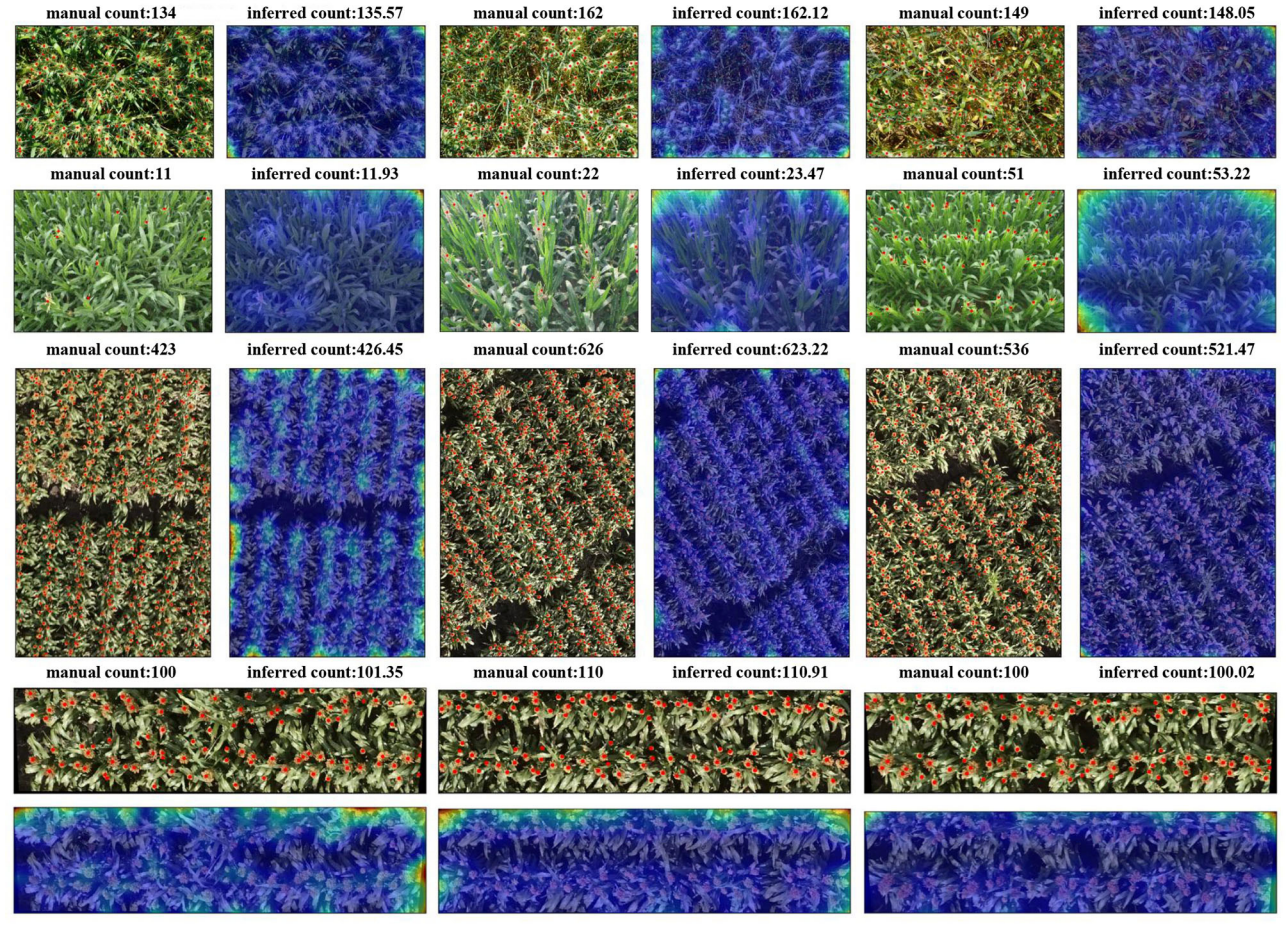
Qualitative results of TasselNetV2+ on three plant counting dataset. From top to bottom, the wheat ears detection dataset, maize tassels counting dataset, sorghum heads counting—dataset1, and sorghum heads counting—dataset2. Red points are manual annotations.

Qualitative results of TasselNetV2+ on three plant counting tasks are shown in Figure 10. TasselNetV2+ infers accurate counts with strong/weak responses on plant/non-plant regions. The resulting count map can be an useful auxiliary cue to benefit related tasks such as detection or segmentation. Note that, TasselNetV2+ are applied to these plant counting tasks with the same architecture and almost the same hyper-parameters (we only slightly vary the batch size to ensure the same number of iterations during parameters updating).

**Figure 11:**
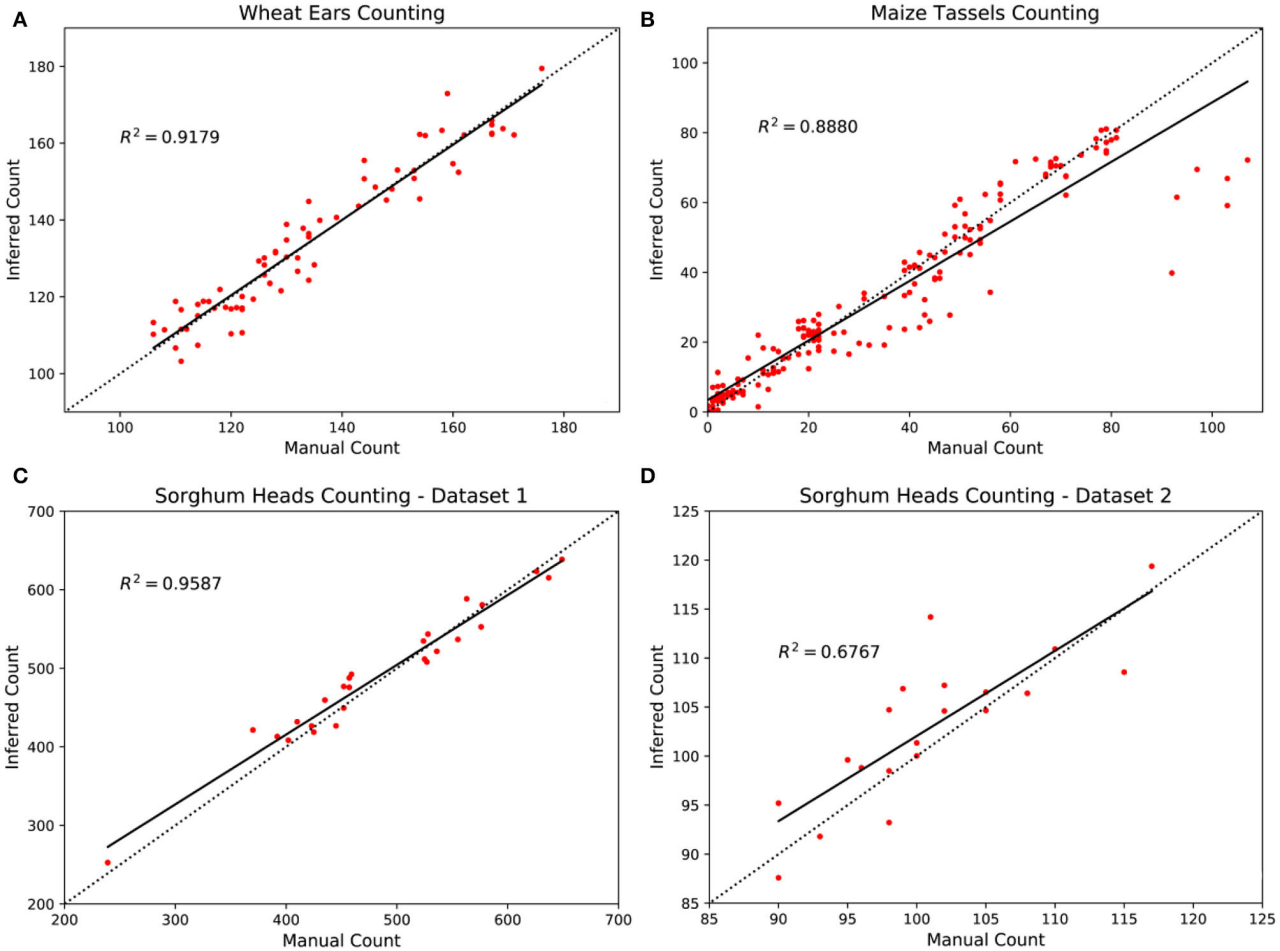
Comparison between inferred counts and manual counts with TasselNetV2+ in four plant counting datasets. **(A)** Wheat ears counting, **(B)** maize tassels counting, **(C)** sorghum heads counting—dataset1, and **(D)** sorghum heads counting—dataset2.

We further compare the manual counts and inferred counts of TasselNetV2+ on three counting tasks in Figure 11. A strong correlation between manual counts and inferred counts is observed on the WED, MTC, and SHC-dataset1 datasets, with *R*^2^ of 0.9179, 0.8880 and 0.9587, respectively. On the SHC-dataset2, the *R*^2^ is slightly poor. We believe the reason is that the points are too sparse such that *R*^2^ can be easily affected by few outliers. Most predictions are sufficiently accurate. We also observe that on the MTC dataset, a set of samples are underestimated. This is because this dataset is the most challenging one with a large data shift between training and testing set. Models learned on the training set may not generalize well to the testing set with significant variations in plant cultivars, illumination changes, and poses. In this case, the idea of domain adaptation may be applied to fill the performance loss (Lu et al., 2017b, 2018).

All evaluation results above suggest the general applicability of TasselNetV2+ in plant counting, especially when only the count value is the output of interest. However, an application note is that, TasselNetV2+ may have limited adaptation to scale variations, e.g., for a model trained on images captured at 5 m height will significantly degrade when testing on images captured at 10 m height. This is because TasselNetV2+ is inherently not a multi-scale model. Fortunately practitioner often have consistent image capturing plans, so this may not be a problem to deploy TasselNetV2+ in reality.

## 4. Conclusion

In high-throughput phenotyping systems, the term “throughput” is closely related to the efficiency of data analysis algorithms. Targeting plant counting, we present TasselNetV2+, a fast implementation of a state-of-the-art plant counting model TasselNetV2, to deal with high-throughput counting from high-resolution imagery. This new implementation is inspired by a time profiling that the computational bottleneck of TasselNetV2 lies in the normalizer. We therefore improve this part with a novel mathematically-equivalent formulation that enables a fast GPU implementation. TasselNetV2+ shows a clear advantage in efficiency on processing high-resolution images. Compared to Faster R-CNN, it also demonstrates its effectiveness and robustness in changed image resolution.

We believe our new implementation will encourage many real-time applications in phenotyping plant counts. An interesting application scenario would be that, images are directly processed right after capturing on the unmanned aircraft vehicles, instead of being sent back for post-processing. It would also be interesting to see applications of TasselNetV2+ to other plant species. For future work, we plan to enhance the scale adaptation of the model.

## Data Availability Statement

All datasets generated for this study are included in the article/supplementary material.

## Author Contributions

HL proposed the idea of TasselNetV2+, implemented TasselNetV2 and TasselNetV2+ in PyTorch, conducted the experiments, analyzed the results, drafted, and revised the manuscript. ZC provided the funding and supervised the study. All authors contributed to the article and approved the submitted version.

## Conflict of Interest

The authors declare that the research was conducted in the absence of any commercial or financial relationships that could be construed as a potential conflict of interest.

## Footnotes

^1^The MATLAB implementation of TasselNetV2 can be found at https://tinyurl.com/TasselNetV2.
